# Diversity and Effect of Increasing Temperature on the Activity of Methanotrophs in Sediments of Fildes Peninsula Freshwater Lakes, King George Island, Antarctica

**DOI:** 10.3389/fmicb.2022.822552

**Published:** 2022-03-17

**Authors:** Diego M. Roldán, Daniel Carrizo, Laura Sánchez-García, Rodolfo Javier Menes

**Affiliations:** ^1^Laboratorio de Ecología Microbiana Medioambiental, Facultad de Química, Universidad de la República, Montevideo, Uruguay; ^2^Laboratorio de Microbiología, Unidad Asociada del Instituto de Química Biológica, Facultad de Ciencias, Universidad de la República, Montevideo, Uruguay; ^3^Centro de Astrobiología, Consejo Superior de Investigaciones Científicas-Instituto Nacional de Técnica Aeroespacial (CSIC-INTA), Madrid, Spain

**Keywords:** Antarctica, global warming, methanotrophs, methane, methane-oxidizing bacteria, methanogenesis, polar lakes

## Abstract

Global warming has a strong impact on polar regions. Particularly, the Antarctic Peninsula and nearby islands have experienced a marked warming trend in the past 50 years. Therefore, higher methane (CH_4_) emissions from this area could be expected in the future. Since mitigation of these emissions can be carried out by microbial oxidation, understanding this biological process is crucial since to our knowledge, no related studies have been performed in this area before. In this work, the aerobic CH_4_ oxidation potential of five freshwater lake sediments of Fildes Peninsula (King George Island, South Shetland Islands) was determined with values from 0.07 to 10 μmol CH_4_ gdw^–1^ day^–1^ and revealed up to 100-fold increase in temperature gradients (5, 10, 15, and 20°C). The structure and diversity of the bacterial community in the sediments were analyzed by next-generation sequencing (Illumina MiSeq) of 16S rRNA and *pmoA* genes. A total of 4,836 ASVs were identified being *Proteobacteria*, *Actinobacteriota*, *Acidobacteriota*, and *Bacteroidota* the most abundant phyla. The analysis of the *pmoA* gene identified 200 ASVs of methanotrophs, being *Methylobacter* Clade 2 (Type I, family *Methylococcaceae*) the main responsible of the aerobic CH_4_ oxidation. Moreover, both approaches revealed the presence of methanotrophs of the classes *Gammaproteobacteria* (families *Methylococcaceae* and *Crenotrichaceae*), *Alphaproteobacteria* (family *Methylocystaceae*), *Verrucomicrobia* (family *Methylacidiphilaceae*), and the candidate phylum of anaerobic methanotrophs *Methylomirabilota*. In addition, bacterial phospholipid fatty acids (PLFA) biomarkers were studied as a proxy for aerobic methane-oxidizing bacteria and confirmed these results. Methanotrophic bacterial diversity was significantly correlated with pH. In conclusion, our findings suggest that aerobic methanotrophs could mitigate *in situ* CH_4_ emissions in a future scenario with higher temperatures in this climate-sensitive area. This study provides new insights into the diversity of methanotrophs, as well as the influence of temperature on the CH_4_ oxidation potential in sediments of freshwater lakes in polar regions of the southern hemisphere.

## Introduction

Climate change, defined as the systematic increase in the average surface temperature of the Earth (Hartmann et al., 2013) has its most marked consequences in polar regions since they suffered an acceleration in their warming compared to other areas of the planet (Pachauri and Meyer, 2014). Particularly, the Antarctic Peninsula and nearby islands have experienced a marked warming trend in the past 50 years. Methane (CH_4_) is one of the most important greenhouse gases [84 times more potent than carbon dioxide over a period of 20 years (Pachauri and Meyer, 2014)] and constitutes an important factor in climate change. It is responsible for approximately 20% of the warming induced by long-lasting greenhouse gases since pre-industrial times (Kirschke et al., 2013). The rise in air temperature produces larger areas of thawing of permafrost and melting of lake ice covers. Consequently, positive feedback can occur by increasing the methane emissions previously trapped in permafrost due to the availability of more organic matter (Anisimov, 2007; Schuur et al., 2009). Moreover, the formation of water deposits contributes significantly to the flow of atmospheric CH_4_ (Walter et al., 2006, 2007).

The net CH_4_ emission from an ecosystem is the balance between opposing processes: production of CH_4_ by methanogenic archaea and consumption by methanotrophic archaea and bacteria (Murrell and Jetten, 2009). In lakes of cold environments, a seasonal behavior takes place (Martinez-Cruz et al., 2015): during autumn-winter, they remain covered with a thick ice layer and CH_4_ can accumulate underneath, but during spring-summer, as the ice layers melt, CH_4_ be released into the atmosphere. In oligotrophic lakes, where oxygen is available throughout the water column, methane oxidation is carried out mainly by aerobic CH_4_-oxidizing bacteria (MOB), especially at the sediment-water interface (Lidstrom and Somers, 1984; Frenzel et al., 1990). Therefore, their functionality is essential to achieve CH_4_ mitigation before it escapes to the atmosphere (Mayr et al., 2020a). MOB are a unique group that can oxidize CH_4_ (and some also methanol) as the only source of carbon and energy with oxygen as the electron acceptor (Trotsenko and Murrell, 2008). Phylogenetically they belong to three lineages: *Alphaproteobacteria*, *Gammaproteobacteria*, and *Verrucomicrobia* (Hanson and Hanson, 1996; Op den Camp et al., 2009). The methanotrophs belonging to the first two phyla have traditionally been classified into type I and type X (*Gammaproteobacteria*) and type II (*Alphaproteobacteria*) based on their phylogeny, physiology, morphology, and biochemistry. However, many exceptions were found lately, so this classification based on the mentioned criteria was put into question (Knief, 2015). It is known that MOB are sensitive to CH_4_ and oxygen concentrations and this can influence the competition between MOB type I and type II (Hanson and Hanson, 1996). Another process, anaerobic oxidation of CH_4_ (AOM) coupled to nitrate (Haroon et al., 2013) or nitrite as electron acceptors (Ettwig et al., 2008) were described as performed by “*Candidatus* Methanoperedens nitroreducens” and “*Candidatus* Methylomirabilis oxyfera” respectively, belonging to group A of the *Methylomirabilota* phylum. The physiology of others members of *Methylomirabilota* phylum (groups B, C, D, and E) is unknown (He et al., 2015). None of the above microorganisms were isolated as pure cultures yet.

The structure and activity of a methanotrophic community in a certain ecosystem are influenced among others by temperature, pH, CH_4_, oxygen, and nitrogen concentrations (Hanson and Hanson, 1996; Mor et al., 2006; Walter et al., 2006; Chanton et al., 2008). The majority of the well-characterized isolated MOB are mesophiles, with only a few strains isolated from cold habitats in the northern hemisphere (Omelchenko et al., 1993; Berestovskaya et al., 2002; Wartiainen et al., 2006) and only one from Antarctica (Bowman et al., 1997). However, methanotrophic activity associated with type I and type II metabolism has been identified over a wide range of temperatures (Trotsenko and Khmelenina, 2005). In general, MOB-Type II are favored at mesophilic temperatures (Urmann et al., 2009) while MOB-Type I prevail at psychrophilic temperatures (0–10°C) (Börjesson et al., 2004; Liebner and Wagner, 2007; Graef et al., 2011).

Maritime Antarctica has been severely affected by climate change, being the area of fastest increase in air temperature in the last five decades (Quayle et al., 2002; Sato et al., 2021), resulting in the ice melting of vast areas and the retreat of glaciers in an accelerated manner. In summer, small streams and lagoons are generated, fed by snow and glacial ice melting. Thus, sediments in Antarctic lakes can be considered integrators of short-term effects of the recent thermal destabilization, taking into consideration the limited outflow and entrance of surface and underground water that carry particulate material from the surroundings (Malandrino et al., 2009). Few studies of CH_4_ oxidation has been carried out in continental lakes of Antarctica (Galchenko, 1994; Bowman et al., 1997; Trotsenko and Khmelenina, 2005), and it was shown that bacterial oxidation consumed >99% of the CH_4_ existing beneath the ice sheet in Subglacial Lake Whillans that represented a significant methane sink (Michaud et al., 2017). Due to the crucial role of methanotrophic activity in controlling CH_4_ emissions (Hanson and Hanson, 1996), it is important to study the structure and the activity of the methanotrophs to temperature fluctuations, considering a future scenario of higher temperatures. The response of MOB to temperature changes was well described in Arctic lakes and sediments (Walter et al., 2007), but to our knowledge, was not described in maritime Antarctica lake sediments, although some particular lakes could be a source of atmospheric CH_4_ (Menes and Roldán, 2019).

The aim of this study was to evaluate the CH_4_ oxidation potential in response to temperature increase for five lacustrine sediments of the Fildes Peninsula (King George Island, South Shetland Islands). Besides, to get insight into the microbial community structure of the *Bacteria* domain with a focus on methanotrophic bacteria, next-generation sequencing of the 16S rRNA and *pmoA* genes was performed. Furthermore, geochemical and PLFA biomarkers data were used to decipher the relation among the diversity of methanotrophs, CH_4_ oxidation potential and lake sediments physicochemical parameters.

## Materials and Methods

### Study Site and Sample Collection

**FIGURE 1:**
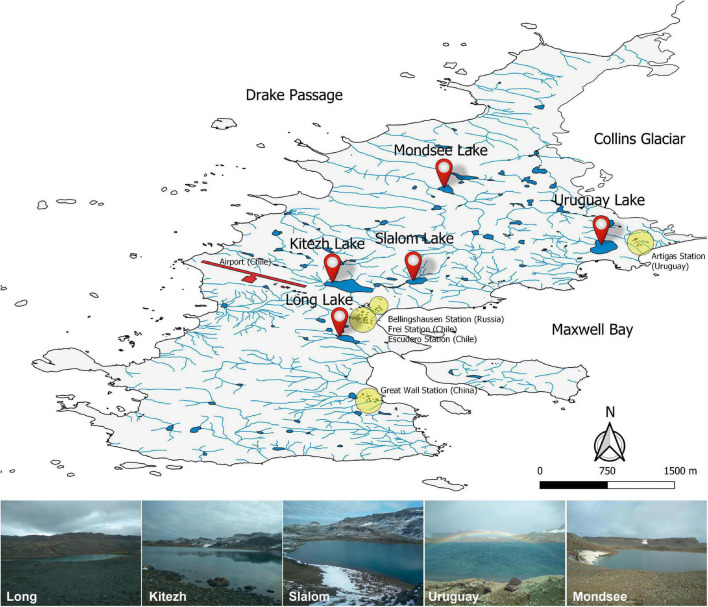
Fildes Peninsula map showing the Antarctic stations, the five studied lakes and illustrative photographs of the sampling sites. The map was modified from PANGAEA (Data Publisher for Earth and Environmental Science, Bremen, Germany. hdl:10013/epic.44322).

The Fildes Peninsula is located in the southwest of King George Island (62°08′–62°14′S and 59°02′–58°51′W), in the South Shetland archipelago, maritime Antarctica. It has a maritime wet climate, with an average annual air temperature of −2.1°C (Michel et al., 2012), with its maximum in summer (daily average 2–3°C), while in winter, the monthly average can fall below −7°C (Braun et al., 2004). Surface sediments (upper 10 cm depth) were collected during the sampling campaign in April 2019 from the deepest area of the lakes by a scuba diver, using sterile plastic tubes. The sediment samples (∼500 g) were transferred to sterile polyethylene containers and refrigerated (4°C) until processed at the laboratory (within 2 weeks). The field campaign supported by the Instituto Antártico Uruguayo (IAU) took place using the Artigas Base (BCAA) in King George Island. The samples were from the following (vernacular names) lakes (Figure 1): Kitezh (62°11′36″S, 58°57′58″W), Long (62°12′18.2″S, 58°57′59″W), Mondsee (62°10′40″S, 58°55′50″W), Slalom (62°11′33″S, 58°57′03″W), and Uruguay (62°11′06″S, 58°54′40″W).

### Physicochemical Analyses

Temperature (*in situ*), pH, and conductivity of the porewater of the sediment samples were measured with an Oakton waterproof pH/CON 450 Meter(Oakton Instruments, Vernon Hills, IL, United States). Inorganic anions and organic acids of low molecular weight were determined by ion chromatography (IC), according to the procedure previously described elsewhere (Parro et al., 2011). In short, 1 g of the sediment samples were sonicated (3 min × 1 min cycles), diluted in 10 mL of deionized water, filtered (22 μm GFF filter), the supernatants were analyzed in a Metrohm 861 Advanced compact ion chromatograph (Metrohm AG, Herisau, Switzerland) with a Metrosep A sup 7-250 column and 3.6 mM sodium carbonate (NaCO_3_) as eluent.

The content of total nitrogen (TN%) and total organic carbon (TOC%) was measured with an elemental analyzer (HT Flash, Thermo Fisher Scientific, Waltham, MA, United States) as described previously (Carrizo et al., 2019).

### Fatty Acids Extraction and Analysis

Extraction of free fatty acids was performed according to the combination of methods described elsewhere (Grimalt et al., 1992; Carrizo et al., 2019). Lyophilized sediments (∼5 g) were spiked with an internal standard (myristic acid-D_27_) and then extracted with a mixture of dichloromethane and methanol (DCM:/MeOH, 3:1, v/v) by ultrasound sonication (3 min × 10 min cycles). The total lipid extract (TLE) of each sample was concentrated to ∼0.5 mL using rotary evaporation and then digested overnight at room temperature in a mixture of methanolic potassium (6% w/w) then separated into neutral and acidic fractions. The neutral lipid fraction was obtained by extracting the methanolic potassium mixture with 30 mL (three times) of *n*-hexane (Hx), rotavaporating, and recovering it with ∼1 mL of Hx:DCM (9:1, v/v). The acidic lipid fraction was obtained by adding HCl (until a pH of 2) to the remaining methanolic potassium mixture and extracting it with 30 mL of Hx (three times), then it was concentrated using rotary evaporation and collected with DCM. The acid fraction was analyzed by gas chromatography mass spectrometry (GC-MS), by injection after derivatization with BF_3_ in MeOH to form fatty acid methyl esters (FAME).

GC-MS analysis was performed using a 6850 GC system coupled to a 5975 VL MSD with a triple axis detector (Agilent Technologies) operating with electron ionization at 70 eV and scanning from *m/z* 50 to 650. Two micro liters of analytes were injected and separated on an HP-5MS column (30 m × 0.25 mm i.d. × 0.25 μm film thickness) with He as a carrier gas at a constant flow of 1.1 mL m^–1^. For analyzing the acidic fraction, the oven temperature was programmed from 70 to 130^°^C at 20°C min^–1^ and then to 300°C at 10°C min^–1^ (held 15 min). The injector temperature was set at 290°C, the transfer line was at 300°C, and the MS source was at 240°C. Compounds identification was based on the comparison of mass spectra with reference materials, and their quantification on the use of external calibration curves of fatty acid methyl esters (FAME; C_8_–C_24_). All chemicals and standards were supplied by Sigma Aldrich (San Luis, Missouri, United States). The recovery of the internal standards averaged 79 ± 14%.

A very distinct characteristic of aerobic methanotrophs is the presence of specific PLFA in the cell membrane that differentiates them from each other (type I have C_16:1ω8*c*_ and C_16:1ω5*t*_ whereas type II have C_18:1ω8*c*_) but also from all other organisms (Bodelier et al., 2009). C_18:1ω7*c*_ was present in some *Methylosinus* species (MOB-Type II) and C_18:2_ (ω7c, 12c, and ω6c,12c) was recognized for its diagnostic value for new members of the family *Methylocystaceae* (MOB-Type II) (Bodelier et al., 2009; Willers et al., 2015). On the other hand, C_16:1ω7*c*_ is associated with members of the *Methylococcaceae* family (MOB-Type I) (Bowman et al., 1993). Consequently, some of these PLFA could be used as a proxy for MOB (Bodelier et al., 2009; Nazaries et al., 2013).

### DNA Extraction, Amplification, Sequencing, and Taxonomic Assignment

DNA extraction was performed from triplicate 0.25g homogenized samples with DNeasy Power Soil kit (Qiagen, Germantown, MD, United States) and stored at −70°C until processing. The amplification and sequencing were performed at Molecular Research MrDNA^1^ (Shallowater, TX, United States), using the Illumina MiSeq sequencing platform, with the following primers 515f-GTGYCAGCMGCCGCGGTAA and 806r-GGACTACNVGGGTWTCTAAT, targeting the variable region V4 of the 16S rRNA gene for *Bacteria* (Caporaso et al., 2011) and A189f-GGNGACTGGGACTTCTGG and mb601r-ACRTAGTGGTAACCTTGYAA for subunit A of the particulate methane monooxygenase gene (*pmoA*) (Costello and Lidstrom, 1999).

For analysis of the 16S rRNA gene sequences, primers and adapters were eliminated. DADA2 was used to infer amplicon sequence variants (ASVs) (Callahan et al., 2016), which was recommended to replace OTU (operational taxonomic unit) based approaches (Callahan et al., 2017; Knight et al., 2018). Sequencing data for the amplicons obtained were processed based on the DADA2 (1.14) analysis pipeline (Callahan et al., 2016) in R (3.6.3) (R Core Team, 2017) with forward, and reverse reads trimmed to 220–200 nt. Reads were truncated when reaching a quality score of 2 and removed if reads contained ambiguous bases, and expected error rate above 2. Then error rates were calculated, filtered reads were dereplicated and the DADA algorithm was utilized to infer exact sequence variants. Forward and reverse reads were merged, and chimeras were removed. 16S rRNA ASVs were taxonomically assigned based on the SILVA database (v138) (Quast et al., 2013). With phyloseq package (1.30.0) (McMurdie and Holmes, 2013), sequences affiliated to mitochondria, chloroplasts, *Archaea* and *Eukarya* were removed and read counts transformed to relative abundance. For *pmoA* gene sequences, primers and adapters were eliminated. Forward and reverse reads trimmed to 270–200 nt, the rest of the pipeline was done using the same configurations as for 16S rRNA analysis with the exception of taxonomical assignment, where modified *pmoA* gene reference database was used (Yang et al., 2016).

### Determination of CH_4_ Oxidation Potential

The sediments were diluted 20% (w/v) with the corresponding lake water in amber glass serum vials in aerobic atmosphere and sealed with butyl rubber stoppers. CH_4_ was then added as a substrate at a concentration of 1% (v/v) of the headspace. The vials were incubated for up to 90 days at 5, 10, 15, and 20°C with constant agitation at 120 rpm in orbital shakers. The experiment was performed in triplicate and blank vials were prepared under the same conditions, but autoclaved (30 min, 121°C) before CH_4_ addition to account for non-biological CH_4_ oxidation. CH_4_ consumption was quantified in the gas phase by periodical sampling and subsequent analysis on a GC-2014 gas chromatograph (Shimadzu Scientific) equipped with a Porapak-Q column (length 6.0 ft, 1/8 in. OD, 2.1 mm ID), column temperature 55°C, detector temperature 140°C, nitrogen as carrier gas (30 mL min^–1^) and a flame ionization detector. CH_4_ oxidation potential rates were determined by linear regression of CH_4_ concentration as a function of time, assuming first-order kinetics.

### Enumeration of Viable CH_4_-Oxidizing Bacteria

Serial dilutions were carried out and viable count were performed by most probable number (MPN) series of three tubes, with nitrate mineral salts medium (NMS) in aerobic atmosphere in Hungate tubes (Whittenbury and Wilkinson, 1970). After sealing the tubes, 1% of CH_4_ was added and tubes were incubated up to 90 days at 5°C. Positive tubes were confirmed by turbidity and CH_4_ consumption in the gas phase as explained above for CH_4_ oxidation potential. The MPN values whose 95% confidence limits do not overlap were considered significantly different (*p* < 0.05).

### Data Analysis

Functions of phyloseq (1.30.0) (McMurdie and Holmes, 2013), vegan (2.5-6) (Oksanen et al., 2018) and ggplot2 (1.30.0) (Wickham, 2006) packages were used for the subsequent analysis and visualization in the R software (3.6.3) of intra and inter-sediment biodiversity at the phylum level, microbial taxa of methanotrophs, rarefaction curves and alpha diversity indices (ASV richness, Shannon diversity and Simpson dominance). To understand which environmental parameters constrain microbial community composition of lake sediments, we performed a non-metric multidimensional scaling (NMDS) ordination analysis based on Bray–Curtis distance and fitted environmental parameters projected by *envfit* function. The NMDS is an unconstrained ordination technique representing multivariate community data in a reduced set of dimensions. Venn diagrams were also constructed to visualize shared and unique ASVs, and microbial taxa between samples for the 16S rRNA and *pmoA* genes. Two variants of Venn diagrams were made, firstly, those that included the total ASVs obtained per sample and secondly, they were grouped with the *tax_glom* function according to the last taxonomic assignment reached by *best_hit*.

Phylogenetic trees were constructed by the neighbor-joining method (Saitou and Nei, 1987) with MOB- and AOM-related ASVs of the 16S rRNA gene, incorporating ASVs from different polar (Arctic and Antarctic) and non-polar (Europe and New Zealand) environments (PRJEB22851 data set) (Kleinteich et al., 2017). The trees for ASVs obtained from the *pmoA* gene were constructed by maximum-likelihood method (Felsenstein, 1981). Trees were generated using the MEGA version 7.0 software package (Kumar et al., 2016). Kimura’s two-parameter model (Kimura, 1980) and Jukes-Cantor model (Erickson, 2010) was used to calculate the evolutionary distance matrices of phylogenetic trees for 16S rRNA and *pmoA* gene, respectively. Bootstrap analysis (1,000 repetitions) was performed to assess the reliability of the branches (Felsenstein, 1985).

The effect of temperature on CH_4_ oxidation potential was evaluated by analysis of variance (ANOVA). The significance of the mean difference was estimated with the Tukey test (*p* < 0.05).

## Results

### Bacterial Diversity

For the 16S rRNA gene, a total of 426,326 sequences grouped in 4,836 ASVs were obtained (Supplementary Table 1), and ranged from 2,355 (Kitezh) to 4,041 (Slalom) (Table 1). Rarefaction curves for 16S rRNA gene sequences showed that the plateau phase was reached for all the lake sediments, with asymptotic tendencies, indicating that the sequencing depth was sufficient to identify the minimum number of reads obtained per sample (Supplementary Figure 1A). The Shannon diversity indices (H’) showed values in the range of 5.92 (Kitezh) to 6.65 (Slalom) and the Simpson dominance indices (D) values from 0.04 (Kitezh) to 0.01 (the other four lakes) (Table 1).

**TABLE 1:** Alpha diversity indices of the five lake sediments based on 16S rRNA and *pmoA* gene analysis.

	Kitezh	Long	Mondsee	Slalom	Uruguay
**16S rRNA gene—bacterial community**					
ASV	2355	3160	3422	4041	2728
H’	5.92	6.60	6.33	6.65	6.05
D	0.04	0.01	0.01	0.01	0.01
**16S rRNA gene—methanotrophs**					
ASV	8	8	9	12	10
H’	1.97	1.58	1.51	2.12	1.89
D	0.16	0.28	0.33	0.15	0.18
***pmoA* gene**					
ASV	20	66	88	106	62
H’	2.07	2.09	2.59	3.34	2.22
D	0.17	0.36	0.27	0.11	0.20

*ASV, Amplicon sequence variant; H’, Shannon index; D, Simpson dominance index.*

Taxonomic assignment revealed a total of 32 phyla, 20 of them with relative abundances higher than 0.05% (Supplementary Figure 2A). In general, the samples showed high abundances of *Proteobacteria* (20.2–30.5%), and minor abundances of *Acidobacteriota*, *Bacteroidota*, *Planctomycetota*, *Verrucomicrobiota*, *Nitrospirota*, *Gemmatimonadota*, *Chloroflexi*, and *Desulfobacterota*. In contrast to the rest of the sediments, Kitezh showed a high abundance of *Actinobacteriota* (34.5%) in relation to *Proteobacteria* (26.1%). In the rest of the sediments, *Actinobacteriota* was present in lower abundances (3.3–11.2%). Remarkably, Uruguay showed a high percentage of *Planctomycetota* (10.2%) and *Chloroflexi* (7.9%) compared to the rest (3.9–6.3% and 2.8–3.9%, respectively). It was also the only sediment that had abundances >0.05% of *Zixibacteria*, *Spirochaetota*, and *Fibrobacterota* (4.6, 3.8, and 3.2% respectively).

In the Venn diagram analysis, the five sediments shared 1,051 ASVs (Supplementary Figure 2B), which represent 21.7% of the total ASVs. Similarly, 11.7% of them were unique, representing 2.0% (Kitezh), 1.3% (Long), 1.1% (Mondsee), 3.7% (Slalom), and 3.6% (Uruguay) of the total ASVs. Moreover, grouping the ASVs according to the last taxonomic affiliation reached (Supplementary Figure 2C) showed a higher percentage of shared microbial taxa among them (52.5%) and fewer percentages of unique taxa (1.3% for Kitezh, 0.9% for Long, 0.4% for Mondsee, 2.3% for Slalom and 1.3% for Uruguay).

A multivariate cluster analysis at a qualitative level (UPGMA, Jaccard index) showed that Long, Mondsee, and Slalom clustered together (Supplementary Figure 3A) separated from Uruguay and Kitezh. Surprisingly, a similar trend was observed when the analysis was performed with the *pmoA* gene (Supplementary Figure 3B).

### Methanotrophs: Diversity, Phylogeny, and Phospholipid Fatty Acids

**FIGURE 2:**
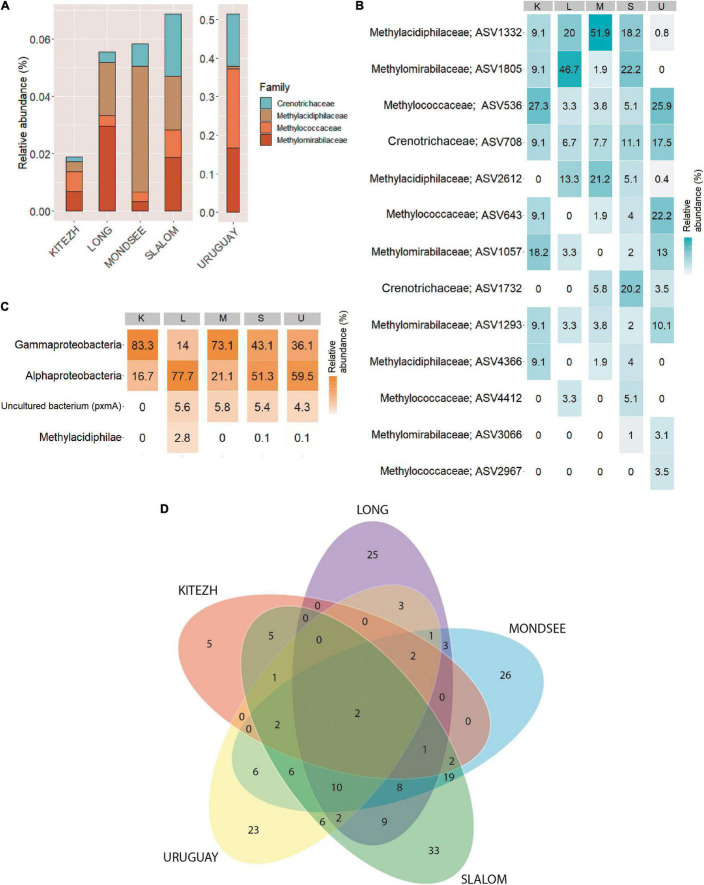
Methane oxidizing bacteria diversity in five lake sediments samples. Relative abundances based on 16S rRNA gene analysis **(A)**. Heatmap showing the percentages of relative abundance of ASVs associated to methane oxidizing bacteria families based on 16S rRNA gene analysis **(B)**, and methane oxidizing bacteria classes based on *pmoA* gene analysis **(C)**. Venn diagram showing number of shared ASVs **(D)**.

Analysis of 16S rRNA gene, focusing on the currently known taxa of MOB and AOM, revealed that only 13 ASVs were identified and in very low relative abundances with respect to the total number of ASVs of the Bacteria domain (Figure 2A): 0.02, 0.06, 0.06, 0.07, and 0.51% (Kitezh, Long, Mondsee, Slalom and Uruguay, respectively). Each sediment had a different abundance distribution of MOB families (Figure 2B): 5.8–39.9%, for *Methylococcaceae* (*Gammaproteobacteria* class), 6.6-31.3% for *Crenotrichaceae* (*Gammaproteobacteria* class), and 1.4–75.0% for *Methylacidiphilaceae* (*Verrucomicrobia* phylum) and 5.8–53.3% for AOM candidate family *Methylomirabilaceae* (*Methylomirabilota* phylum). ASVs affiliated with *Alphaproteobacteria*-MOB were not detected in this analysis.

For the *pmoA* gene, 12,540 sequences grouped in 200 ASVs were obtained (Supplementary Table 2). Rarefaction curves are shown in Supplementary Figure 1B. The analysis showed a different relative abundance distribution for each sediment compared to the 16S rRNA gene (Figure 2C): *Alphaproteobacteria*-MOB ASVs (16.7–77.7%) were identified, some of them reaching the taxonomic assignment of *Methylocystaceae* family. In addition, *Gammaproteobacteria*-MOB ASVs (14.0–83.3%), were also detected, some of them reaching the *Methylococcales* order, and the *Methylococcaceae* family. *Methylacidiphilae*-MOB ASVs (0.1–2.8%) were detected only in Long, Slalom and Uruguay, where the taxonomic assignment reached the genus *Methylacidiphilum*. Finally, a low percentage of ASVs in Long, Mondsee, Slalom and Uruguay (4.3–5.8%) corresponded to non-cultivable environmental *pmoA* sequences.

In the Venn diagram analysis with the *pmoA* gene, the five lake sediments shared only 2 ASVs (Figure 2D), which represent 1.0% of the total identified ASVs. Likewise, 56.0% of total ASVs were unique for each lake sediment, representing 2.5% (Kitezh), 12.5% (Long), 13.0% (Mondsee), 16.5% (Slalom), and 11.5% (Uruguay) of the total ASVs. In addition, grouping the ASVs according to the last taxonomic affiliation reached, showed that a higher similarity among lake sediments was obtained (50% of total methanotrophs), with only Mondsee and Uruguay showing unique taxa (data not shown).

**FIGURE 3:**
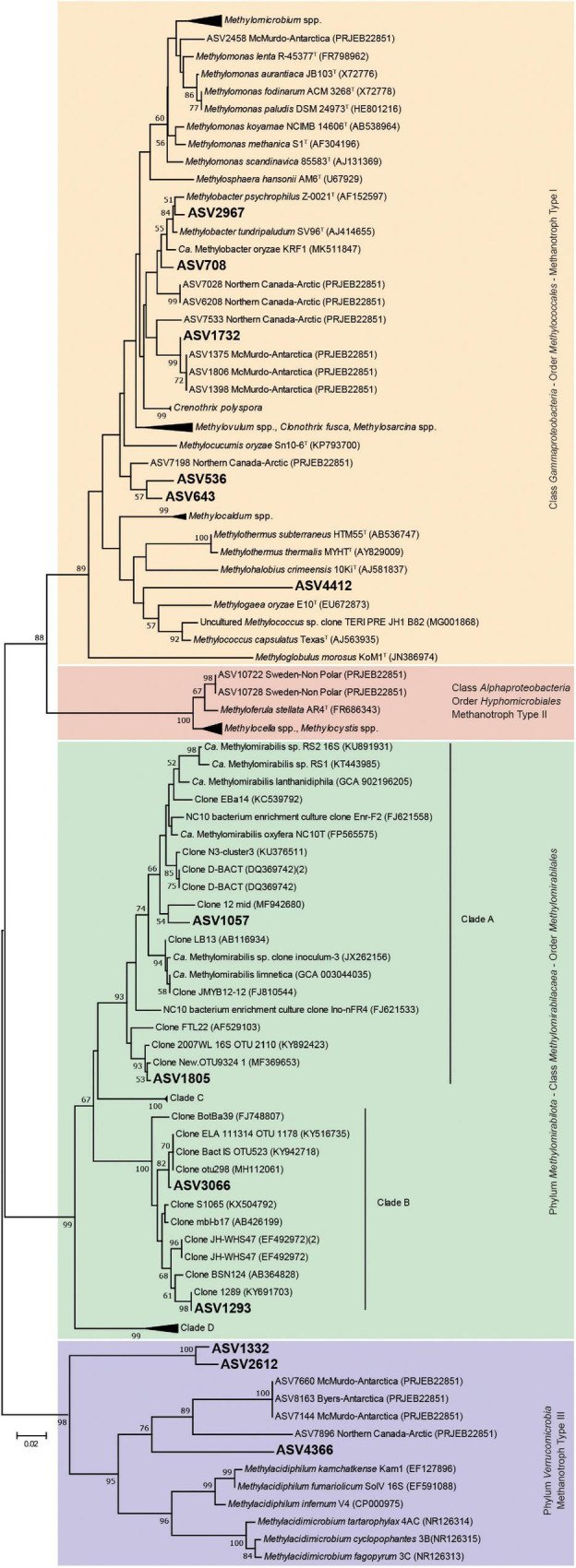
Neighbor-joining tree of partial bacterial 16S rRNA gene sequences and representative species of currently know aerobic methane oxidizing bacteria and anaerobic methane oxidizing bacteria of the *Methylomirabilota* phylum. Kimura’s two parameter model was used to calculate the evolutionary distance matrices based on 253 nucleotide positions. Bootstrap values (above 50%) based on 1,000 re-samplings are listed at the nodes. Bar, 0.02 substitutions per nucleotide position.

The phylogenetic analysis of the ASVs affiliated with methanotrophs based on 16S rRNA gene (Figure 3), showed that the majority were highly related to MOB-Type I (*Gammaproteobacteria* class). ASV2967 and ASV708 joined the clade of *Methylobacter psychrophilus*, *Methylobacter tundripaludum* (both psychrophilic species), and “*Candidatus* Methylobacter oryzae,” which constitute a cluster integrated by members of the environmentally important *Methylobacter* Clade 2 (Smith et al., 2018). ASV708 was detected in all sediments with the highest relative abundance of methanotrophs (6.7–17.5%) (Supplementary Table 1).

ASV1732 was related to *Crenothrix polyspora*, a filamentous bacterium that has been reported as one of the main consumers of CH_4_ in stratified lakes (Oswald et al., 2015, 2016, 2017). However, it clustered in a distinct clade within *Crenothrix*-related ASVs from Antarctic and Arctic environments, separated from *Crenothrix polyspora* clone sequences. ASV536, ASV643, and ASV4412, joined a cluster separated from other known MOB-Type I (*Gammaproteobacteria* class), indicating that represent presumably novel species of MOB not yet characterized. ASV536 and ASV643 (close to *Methylocucumis oryzae*) were abundant in Uruguay lake sediments, which also showed a significantly higher methanotrophic activity than the other sediments. ASV1332, ASV2612, and ASV4366 joined a cluster within the family *Methylacidiphilaceae* (phylum *Verrucomicrobia*), but far distant and separated from the group of acidophilic and thermophilic MOB *Methylacidiphilum infernum, Methylacidiphilum fumariolicum*, and *Methylacidiphilum kamchatkense*, and acidophilic and mesophilic MOB *Methylacidimicrobium tartarophylax*, *Methylacidimicrobium. cyclopophantes*, and *Methylacidimicrobium fagopyrum*. In particular, ASV4366 clustered with the rest of *Verrucomicrobia*-related ASVs identified in polar (Arctic and Antarctic) environments. ASV1057, ASV1805, ASV1293, and ASV3066 joined the family *Methylomirabilaceae*. However, differences were found in their similarities: the first two are close to group A, and the last two to group B (Ettwig et al., 2009; He et al., 2015).

On the other hand, the phylogenetic analysis of ASVs based on *pmoA* gene (Supplementary Figure 4) showed that few ASVs were affiliated with any known taxa. Regarding the *Gammaproteobacteria* class, ASV142 and ASV165 clustered with “*Candidatus* Methylobacter oryzae,” ASV67 with *Methylobacter capsulatum*, ASV185 with *M. psychrophilus*, ASV67 with *Methylococcus capsulatus*, and ASV166 and ASV7 with *Methylomonas paludis*. In the *Alphaproteobacteria* class, ASV183 clustered with *Methylosinus acidophilus*. Other related ASVs (ASV1, ASV13, ASV17, ASV52, ASV94 and ASV132) clustered in the clade that also harbors *M. acidophilus*. However, the affiliation of these ASVs was uncertain. Finally, none of the ASVs clustered within known taxa in the *Verrucomicrobia* phylum.

Phospholipid fatty acids biomarkers analysis showed a wide variety of fatty acids, those with potential biomarker value for characterized MOB were selected (Supplementary Table 3). Regarding fatty acids of MOB-Type I, all the lakes presented the fatty acid C_16:1ω7*c*_ in concentrations from 0.05 ng gdw^–1^ (Kitezh), and from 1.96 ng gdw^–1^ to 6.05 ng gdw^–1^ (the other sediments). C_18:2ω6,12*c*_ (MOB-Type II), were also present in all sediments in concentrations from 0.11 ng gdw^–1^ (Kitezh) to 2.15 ng gdw^–1^ (Uruguay). Uruguay also showed the presence of C_18:1ω7*c*_, characteristic of some *Methylosinus* species (also present in Long) and C_18:1ω8*c*_ (MOB-Type II) with concentrations of 2.66 ng gdw^–1^ and 1.35 ng gdw^–1^, respectively.

### Relationships Between Environmental Parameters and Microbial Diversity

**FIGURE 4:**
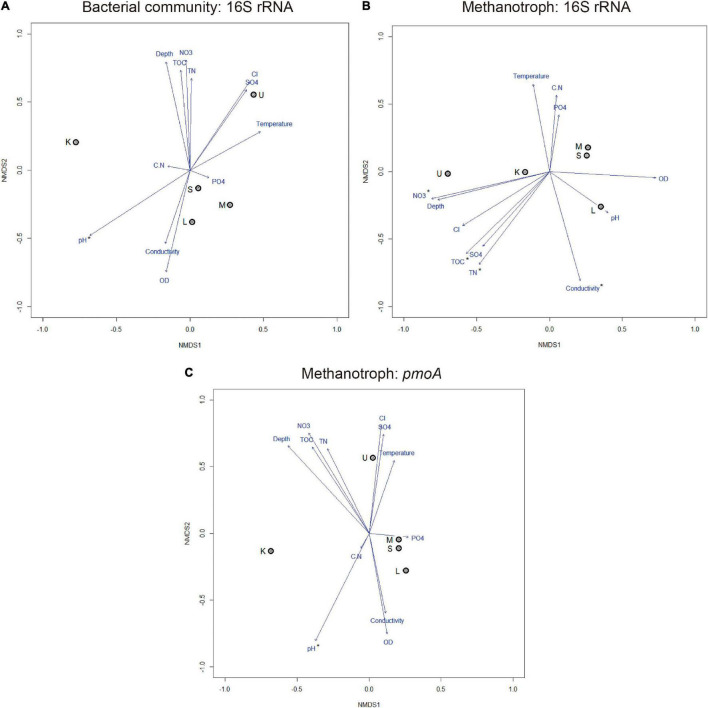
Non-metric multidimensional scaling ordination plots based on Bray–Curtis distance and environmental variables showing the relationships among samples based on total bacterial community composition **(A)** and methanotrophs **(B,C)**, stress < 0.20. Arrows with asterisks indicate a significant correlation (*p* < 0.05). DO, dissolved oxygen; TOC, total organic carbon; TN, total nitrogen; CN, TOC/TN ratio; PO4, phosphate; SO4, sulfate; Cl, chloride; NO3, nitrate. K, Kitezh; L, Long; M, Mondsee; S, Slalom; and U, Uruguay.

The result of physicochemical analyses is shown in Table 2. The NMDS analysis (Figure 4) showed that among the 12 evaluated physicochemical parameters, the pH correlated with the composition of the total bacterial community (16S rRNA gene) and with methanotrophs (*pmoA* gene) (*r*^2^ = 0.99, *p* < 0.05; *r*^2^ = 0.99, *p* < 0.001, respectively). In particular, the subset of methanotrophs evaluated by 16S rRNA gene correlated with TOC (*r*^2^ = 0.98, *p* < 0.001), total nitrogen (TN, *r*^2^ = 0.99, *p* < 0.001), conductivity (*r*^2^ = 0.98, *p* < 0.05), and nitrate concentration (NO_3_, *r*^2^ = 0.98, *p* < 0.05).

**TABLE 2:** General data and physicochemical characteristics for the five freshwater lake sediments.

	Kitezh	Long	Mondsee	Slalom	Uruguay
Latitude	62°11′36″S	62°12′18″S	62°10′40″S	62°11′33″S	62°11′06″S
Longitude	58°57′58″W	58°57′59″W	58°55′50″W	58°57′03″W	58°54′40″W
Depth (m)	15.0	4.0	4.0	4.0	17.0
Temperature (°C)	4.8	4.0	7.4	5.4	6.7
DO (mg L^–1^)	11.99	12.50	12.80	11.77	10.65
pH	7.96	7.38	6.97	7.09	5.88
Conductivity (μS cm^–1^)	185	303	162	150	128
TOC (%dw)	3.62	2.10	0.51	0.97	5.20
TN (%dw)	0.25	0.20	0.04	0.05	0.42
C/N	14	11	13	19	12
Acetate	Nd	Nd	0.02	0.09	0.57
Propionate	0.11	Nd	0.05	0.20	3.86
Formate	0.16	0.10	0.11	0.18	1,76
Phosphate	Nd	Nd	Nd	0.12	0.02
Sulfate	1.44	5.68	1.39	0.84	14.88
Chloride	0.94	3.61	1.57	0.74	15.28
Nitrite	Nd	Nd	Nd	Nd	Nd
Nitrate	0.03	Nd	Nd	Nd	0.05
Bromide	Nd	Nd	Nd	Nd	Nd
Fluoride	Nd	Nd	Nd	Nd	Nd

*DO, dissolved oxygen. TOC and TN, total organic carbon and total nitrogen respectively (percentages are relative to total dry weight). C/N, ratio of TOC over TN (dimensionless). Concentration of inorganic and organic anions expressed in μg per g of dry weight. Nd, not detected.*

### CH_4_ Oxidation Potential and CH_4_-Oxidizing Bacteria Viable Count

**FIGURE 5:**
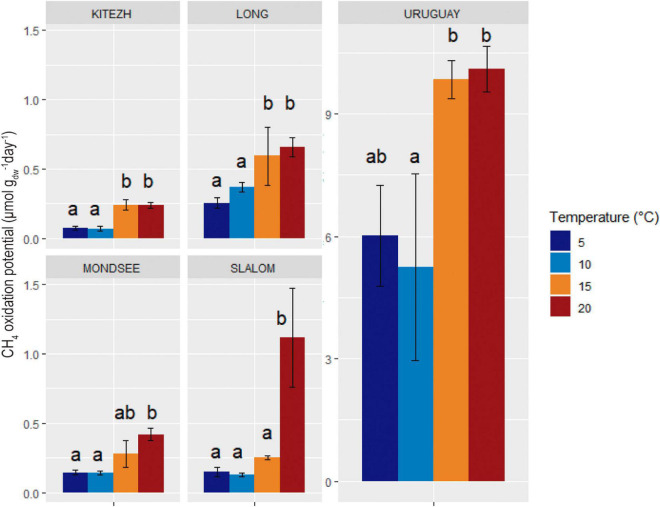
Influence of temperature on CH_4_ oxidation potential in five lake sediments samples. Errors bars of standard deviation of triplicate runs are indicated. Different letters indicate significant differences (Tukey, *p* < 0.05).

All evaluated sediments had measurable CH_4_ oxidation activity at every temperature (Figure 5). The values ranged from 0.07 to 10 μmol CH_4_ gdw^–1^ day^–1^ and Uruguay had the highest values at all temperatures. The CH_4_ oxidation potential values obtained at 5 and 10°C were not significantly different for each lake sediment. At 5°C (close to the *in situ* lake temperatures), no significant differences were found among Kitezh, Long, Mondsee, and Slalom lake sediments. An increase of CH_4_ oxidation potential with temperature was observed for all sediments, with the highest CH_4_ consumption values around 20°C or higher.

The MOB count values calculated by MPN for Kitezh, Long, Mondsee, and Slalom were very low and not significantly different (Table 3). On the other hand, Uruguay showed a count value of two orders of magnitude higher compared to the other sediments.

**TABLE 3:** Methanotrophic oxidizing bacteria viable count and 95% confidence limits for the five lake sediments.

	MPN/g	Lower limit	Upper limit
Kitezh	3.6	0.17	18
Long	9.2	1.4	38
Mondsee	15	3.7	42
Slalom	29	8.7	94
Uruguay	1,100	180	4,100

## Discussion

### Bacterial Diversity

Microorganisms dominate the genetic pool and biomass of colonizable niches in most Antarctic environments, playing key roles to maintain ecosystem functions. The main bacterial phyla found in the five sediments were similar to those found in sediments and soils of polar environments (Sjöling and Cowan, 2003; Saul et al., 2005; Smith et al., 2006; Achberger et al., 2016; Koo et al., 2018). The roles of these groups of microorganisms in sediments are associated with essential ecosystem functions, from primary production, heterotrophy, and recycling of minerals (Wilkins et al., 2013). Although a literature review showed that freshwater deposits in the region are very homogeneous, the differences in the abundance of certain taxa may be due to anthropogenic impact or the contribution of exogenous organic matter in some lakes (Saul et al., 2005; Laybourn-Parry and Pearce, 2007; Ransom-Jones et al., 2012). The diversity values found in this work were similar to other lake systems in cold environments (Christner et al., 2014; Wang et al., 2016; Liao et al., 2019; Broman et al., 2020). According to the results obtained, the five sediments had balanced communities, without great dominance.

It is well-known that environmental factors can affect the abundances and taxonomic compositions of microbial communities and some studies have shown that pH was found to be the dominating factor driving the variations in community structure, as in our study (Xiong et al., 2012; Wilhelm et al., 2013; Ligi et al., 2014; Liu et al., 2015; Wang et al., 2016).

### Methanotrophs: Diversity and Phylogeny

According to the 16S rRNA and *pmoA* gene results, MOB-Type I and MOB-Type II were both present in all sediments with relative abundances in the range as previously reported in Arctic and subarctic lakes (He et al., 2012a). In our study, some taxa of methanotrophs were not detected in the analysis of the 16S rRNA gene sequencing and could be attributable to the low abundance of these microorganisms relative to total Bacteria. It has been shown that single-gene surveys fail to capture the taxonomic and metabolic diversity of these microorganisms (Mayr et al., 2020b). Thus, the combination of target genes is considered one of the best approaches to reveal methanotrophic diversity (Lüke and Frenzel, 2011). We applied amplicon sequencing of *pmoA* gene with primers A189f and mb601r, which was reported as one of the best pair of primers for the coverage of methanotrophs diversity (Bourne et al., 2001; McDonald et al., 2008). However, it is necessary to emphasize that the primers used may not be able to amplify all MOB as well as AOM sequences of the *Methylomirabilota* phylum (Luesken et al., 2011). Therefore, since the *pmoA* primers used have some limitations, an analysis that combines also 16S rRNA gene primers is advantageous, allowing a more complete approximation of the structure of the methanotrophic community in this unexplored environment.

The reduced biodiversity of methanotrophs found in these sediments makes them ideal model systems to examine the effect of factors such as temperature and geochemistry parameters. Consequently, this particular process could be an indicator of the impact of those factors.

Fatty acids (C_16:1ω7*c*_) specific of the *Methylococcaceae* family were identified in all samples, in agreement with the high relative abundance of ASVs affiliated to *Methylobacter* Clade 2. In addition, fatty acids (C_18:2ω6,12*c*_) associated MOB-type II *Alphaproteobacteria* were also present in all lakes. However, some of these fatty acids are not exclusive of methanotrophs and are distributed in other taxonomic groups (Willers et al., 2015). Consequently, the detection of these fatty acids was carried out as complementary confirmation of the presence of different groups of MOB in these sediments, along with the sequencing data (Bodelier et al., 2009; Nazaries et al., 2013). In addition, great care should be taken in interpreting these data since the PLFA database for methanotrophs (especially psychrophiles) is not extensive (McDonald et al., 2008).

When considering the sequencing data and PLFA results together, the presence of both groups of MOB in the sediments can be assumed. It was observed that those lakes that have a higher relative abundance of the MOB-Type II associated *pmoA* gene, showed a higher concentration of fatty acids C_18:2ω6_, C_18:1ω7c_, and C_18:1ω8*c*_.

These preliminary results present a future perspective for the analysis of these communities by SIP-PLFA in Antarctic environments, and confirm which fatty acids are characteristic of these particular methanotrophs in the lake sediments studied. In southern polar environments, where information on MOB is scarce, the approach to these communities based on the composition of PLFA can reveal novel data that broaden the knowledge of cell membranes of psychrophilic or psychrotolerant microorganisms. Given that currently, the sequences of cultivated psychrophilic methanotrophic bacteria are scarce, the identification of species based on sequence similarity is biased in cold environments. In our study, the assignment of some ASVs to the *Methylacidiphilaceae* family was unexpected, since their cultivated representatives described so far were mainly associated to geothermal environments, with high temperatures and low pH (Op den Camp et al., 2009; van Teeseling et al., 2014). However, the new description of mesophilic representatives (van Teeseling et al., 2014) and the results obtained in this research, suggest a broader phylogenetic spectrum of MOB of the phylum *Verrucomicrobia*, occupying several environments, fulfilling an ecological role not yet clearly clarified.

ASV708, ASV2967 (16S rRNA gene), ASV142, and ASV165 (*pmoA* gene) were clearly affiliated to *Methylobacter* Clade 2. ASV1732 (this study) and other Arctic and Antarctic sediments ASVs (Kleinteich et al., 2017) were affiliated to a different cluster that could also be members of the *Methylobacter* Clade 2, given the lack of isolates that expand the knowledge of this group. Members of this clade have usually been related to cold-adapted niches (*M. tundripaludum* and *M. psychrophilus*) (Smith et al., 2018). However, new members associated with other environments have recently been discovered, e.g., “*Ca.* Methylobacter oryzae” KRF1, isolated from tropical rice fields (Rahalkar et al., 2019; Khatri et al., 2020). This organism has genes coding for dissimilatory nitrate reduction pathway (Smith et al., 2018), an ability that according to Hernandez et al. (2015) would allow them to survive under hypoxic conditions or be more competent against other type I methanotrophs. *Methylobacter* Clade 2 has been reported as the dominant methane oxidizer in soils and sediments, becoming an active cosmopolitan methanotroph present in many ecosystems (Kleinteich et al., 2017; Rahalkar et al., 2019; Khatri et al., 2020). The association of ASV536 and ASV643 with the closest taxon *Methylocucumis oryzae* (Pandit et al., 2018; Pandit and Rahalkar, 2019), and the aforementioned relationship with “*Ca.* Methylobacter oryzae” adds information on the groups of methanotrophs studied and their wide distribution throughout different environments on the planet, including in Antarctic lake sediments.

Members of the *Methylomirabilaceae* family (whose best-characterized representative is “*Ca.* Methylomirabilis oxyfera”) has been reported as dominant in anaerobic enrichments with CH_4_ as substrate (Wu et al., 2011; Cui et al., 2015), suggesting that in the studied sediments, members represented by ASV1805 and ASV1057 would be carrying out the same metabolic activity. Although ASV1293 and ASV3066 were related to the *Methylomirabilota* phylum, it cannot be assured that they were AOM representatives since there is no member of this group that has been enriched or isolated, and their ecophysiology is unknown (Ettwig et al., 2009; He et al., 2015). The identification of AOM in the ASVs of the *Methylomirabilaceae* family raises the possibility of CH_4_ oxidation processes associated with denitrification in the anoxic phase of these sediments (Cui et al., 2015). These bacteria would consume the CH_4_ generated by methanogenic archaea prior to reaching the oxic phase, where it would be oxidized by MOB before it would be released into the atmosphere. The results obtained open the question of including these taxa in the biogeochemical cycle of CH_4_ in maritime Antarctic lake sediments in future studies. Moreover, since our molecular tools did not cover anaerobic methanotrophic archaea, their influence on the methane cycle cannot be overlooked.

It has been postulated that most Antarctic prokaryotes diverged from their closest known non-antarctic relatives long before geographic isolation developed in the Antarctic region (Franzmann, 1996). Therefore, the evolution processes by isolation could have given rise to communities of microorganisms endemic of this region (Tindall, 2004), as psychrophilic or psychrotolerant methanotrophic organisms, with possible new genera and species. This would explain the presence of presumably new microbial clades of methanotrophic bacteria in these sediment samples. Particularly, the *pmoA* gene sequencing results showed a large number of ASVs that did not reach the taxonomic rank of known family or genus, or the ASVs of 16S rRNA gene related to the phylum *Gammaproteobacteria* type I, but far from known methanotrophic species, as well as MOB of the phylum *Verrucomicrobia*. The low biodiversity of MOB could reflect a functional disadvantage of this ecosystem since it is expected that redundancy in more diverse communities would stabilize their functioning (Bell et al., 2005).

### CH_4_ Oxidation Potential and CH_4_-Oxidizing Bacteria Viable Count

The values of CH_4_ oxidation potentials increased with temperature from 5 to 15°C. However, for Kitezh, Long, Mondsee, and Uruguay there were no significant differences at 15 and 20°C, suggesting that active MOB were psychrotolerant (with an optimum temperature between 15 and 20°C) and not real psychrophiles (Morita, 1975). On the other hand, for Slalom, active MOB seemed to have optimal temperatures close to mesophilia (with an optimum temperature around or higher than 20°C). Some studies carried out on samples from northern polar regions revealed that the optimum temperature for methanotrophic activity was between 15 and 25°C (Martineau et al., 2010; He et al., 2012b), and some of them even showed activity at 38°C (Liebner and Wagner, 2007). Therefore, our results agree with previous approaches where they concluded that many microorganisms can survive and multiply in extreme conditions imposed by low-temperature environments, far from their optimum temperature (Rothschild and Mancinelli, 2001). The pressure of low temperatures in maritime Antarctica could lead to the selection of psychrotolerant microorganisms, whereas MOB-Type I (associated with lower optimum temperatures) tend to outgrow MOB-Type II (Börjesson et al., 2004; Liebner and Wagner, 2007). However, the latter would prevail in oligotrophic environments. Consequently, both types of methanotrophs could carry out the ecological role and structure the community of MOB depending on the environmental conditions and its spatial distribution (Mayr et al., 2020b).

Since the information on methanotrophic activity and diversity in these regions is scarce, it was not possible to compare our results with other similar studies. Comparing CH_4_ oxidation potential values for Kitezh, Long, Mondsee, and Slalom they were in the range of other studies in northern polar regions (Liebner and Wagner, 2007; Martineau et al., 2010; He et al., 2012a,b; Mo et al., 2020).

The positive influence of temperature on the production of CH_4_ in sediments of this region was previously verified in microcosm tests (Ellis-Evans, 1984; Vieira et al., 2015; Menes and Roldán, 2019), consequently, it was important to verify that these sediments could also have the capacity to mitigate CH_4_ emissions at higher temperatures.

The results of the enumeration of MOB indicated low numbers of culturable bacteria in the sediments, 10^3^–10^6^ times lower than the results of viable counts in sediments of Ace Lake and Burtoon Lake in East Antarctica (Bowman et al., 1997). It was also 10^2^–10^6^ times lower than the values from sediments of other polar regions (Trotsenko and Khmelenina, 2005; Liebner and Wagner, 2007; Oshkin et al., 2014) obtained by quantification of individual cells by independent culture methods (immunofluorescence and FISH). Viable count techniques difficulties for this group of microorganisms are fully recognized (McDonald et al., 2008) since they are highly affected by the type of medium selected and the incubation conditions. However, our results are consistent with the low relative abundance of MOB found by the 16S rRNA gene analysis. In lakes of East Antarctica, it has been shown that methane oxidation rate correlated well with the number of methanotrophs (Galchenko, 1994) as in our study, where the sediment that showed a higher count presented higher methanotrophic activity. In addition, the PFLA analysis showed a positive trend with the potential oxidation activity and MPN results, since the sediment with the highest activity and viable count (Uruguay), showed the highest concentration of MOB-related fatty acids, in contrast to the one with the lowest activity and count (Kitezh), which showed the lowest concentration.

## Conclusion

The lacustrine sediments studied had a balanced bacterial diversity without great dominance. In particular, the methanotrophic bacteria were in low abundance and had low biodiversity, suggesting a high vulnerability of this population that deserves particular attention.

Our results also indicate a high similarity of methanotrophic communities across lakes. According to the most abundant ASVs in all sediments, it can be concluded that aerobic methanotrophy is mainly due to members of the *Methylobacter* Clade 2 (Type I family *Methylococcaceae*). The results obtained for the CH_4_ oxidation potential revealed values capable of mitigating CH_4_ emissions and a favorable influence of temperature up to 20^°^C. This study opens up interesting perspectives to further deepen the knowledge of methanotrophy in maritime Antarctica, such as isolating new methanotrophic species and having a better understanding of the methanotrophy in aerobiosis as well as in anaerobiosis. Our results set a starting point to delve deeper into the knowledge of the biogeochemical cycle of CH_4_ in maritime Antarctica.

## Data Availability Statement

The datasets presented in this study can be found in online repositories. The names of the repository and accession number(s) can be found below: https://www.ncbi.nlm.nih.gov/bioproject/PRJNA637149.

## Author Contributions

DR and RM designed the study and experimental details. DR conducted the experiments, completed the analysis, and wrote the initial draft. RM, DC, and LS-G contributed to the manuscript and editing. All authors contributed to the article and approved the submitted version.

## Conflict of Interest

The authors declare that the research was conducted in the absence of any commercial or financial relationships that could be construed as a potential conflict of interest.

## Publisher’s Note

All claims expressed in this article are solely those of the authors and do not necessarily represent those of their affiliated organizations, or those of the publisher, the editors and the reviewers. Any product that may be evaluated in this article, or claim that may be made by its manufacturer, is not guaranteed or endorsed by the publisher.

## Footnotes

1
www.mrdnalab.com
