# Supplementary material for: Diversity and Effect of Increasing Temperature on the Activity of Methanotrophs in Sediments of Fildes Peninsula Freshwater Lakes, King George Island, Antarctica
Source: Front Microbiol. 2022 Mar 17;13:822552. doi: 10.3389/fmicb.2022.822552 (PMC8969513; doi:10.3389/fmicb.2022.822552)

Supplementary Material

Diversity and Effect of Increasing Temperature on the Activity of Methanotrophs in Sediments of Fildes Peninsula Freshwater Lakes, King George Island, Antarctica

Diego M. Roldán, Daniel Carrizo, Laura Sánchez-García and Rodolfo Javier Menes

1 Supplementary Tables

Table S3. Phospholipid fatty acids biomarkers of aerobic methanotrophs for five freshwater lake sediments of Fildes Peninisula, Antarctica (ng g^{-1} of dry weight). Nd, Not detected.

2 Supplementary Figures

Figure S1: Rarefaction curves for **(A)** 16S rRNA gene (total Bacteria) and **(B)** *pmoA* gene (methanotrophs) for five samples of freshwater lake sediments

Figure S2: Bacterial diversity based on 16S rRNA gene analysis in five lake sediments samples

Figure S3: Dendrogram obtained by clustering analysis with UPGMA algorithm, using Jaccard index for similarity measures (height values in the scale)

Figure S4: Maximum likelihood tree of partial bacterial *pmoA* gene sequences and representative species of currently know methane oxidizing bacteria of *Gammaproteobacteria* class **(A)**, *Alphaproteobacteria* class **(B)** and *Verrucomicrobia* phylum **(C)**.

Supplementary Table S3. Phospholipid fatty acids biomarkers of aerobic methanotrophs for five freshwater lake sediments of Fildes Peninisula, Antarctica (ng g^{-1} of dry weight). Nd, Not detected.

	Kitezh	Long	Mondsee	Slalom	Uruguay
$C_{16:1\omega7c}$	0.05	4.01	1.96	6.05	4.58
$C_{18:2\omega6,12c}$	0.11	0.43	0.17	0.41	2.15
$C_{18:1\omega7c}$	Nd	0.17	Nd	Nd	2.66
$C_{18:1\omega8c}$	Nd	Nd	Nd	Nd	1.35

Supplementary Figure S1. Rarefaction curves for 16S rRNA gene (total Bacteria) **(A)** and *pmoA* gene (methanotrophs) **(B)** for five samples of freshwater lake sediments. K = Kitezh, L = Long, M = Mondsee, S = Slalom and U = Uruguay

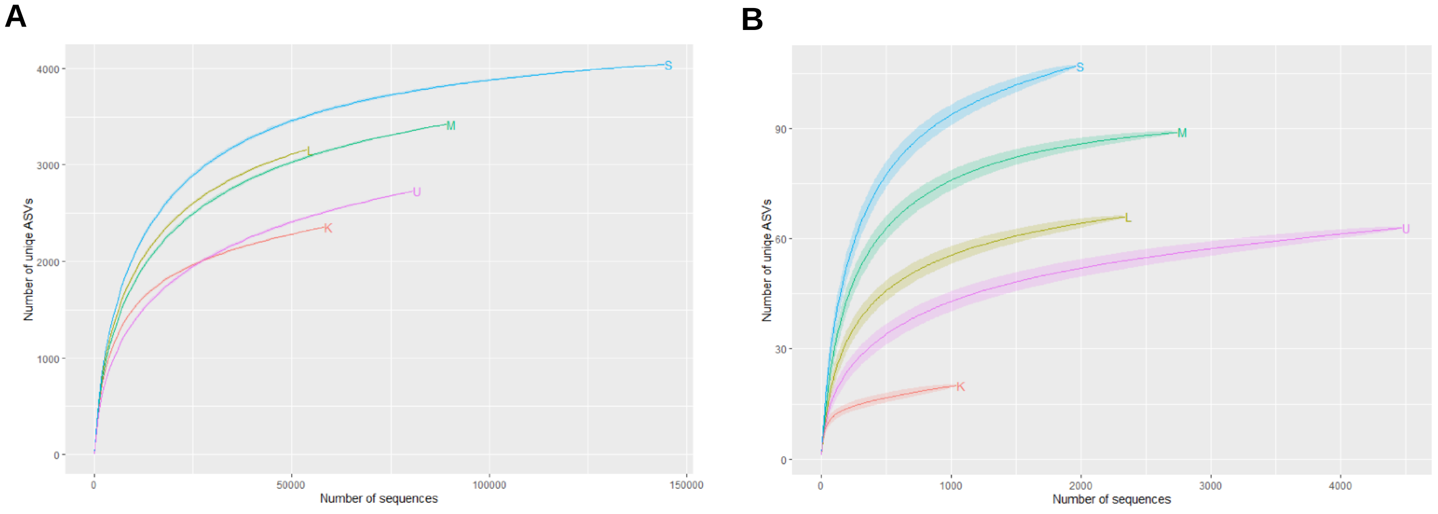

Supplementary Figure S2. Bacterial diversity based on 16S rRNA gene analysis in five lake sediments samples. Heatmap showing the percentages of relative abundance of the 32 phyla present in the samples **(A)**. Venn diagram showing number of shared ASVs **(B)** and shared microbial taxa agglomerated by the lower taxonomic rank assignment **(C)**.

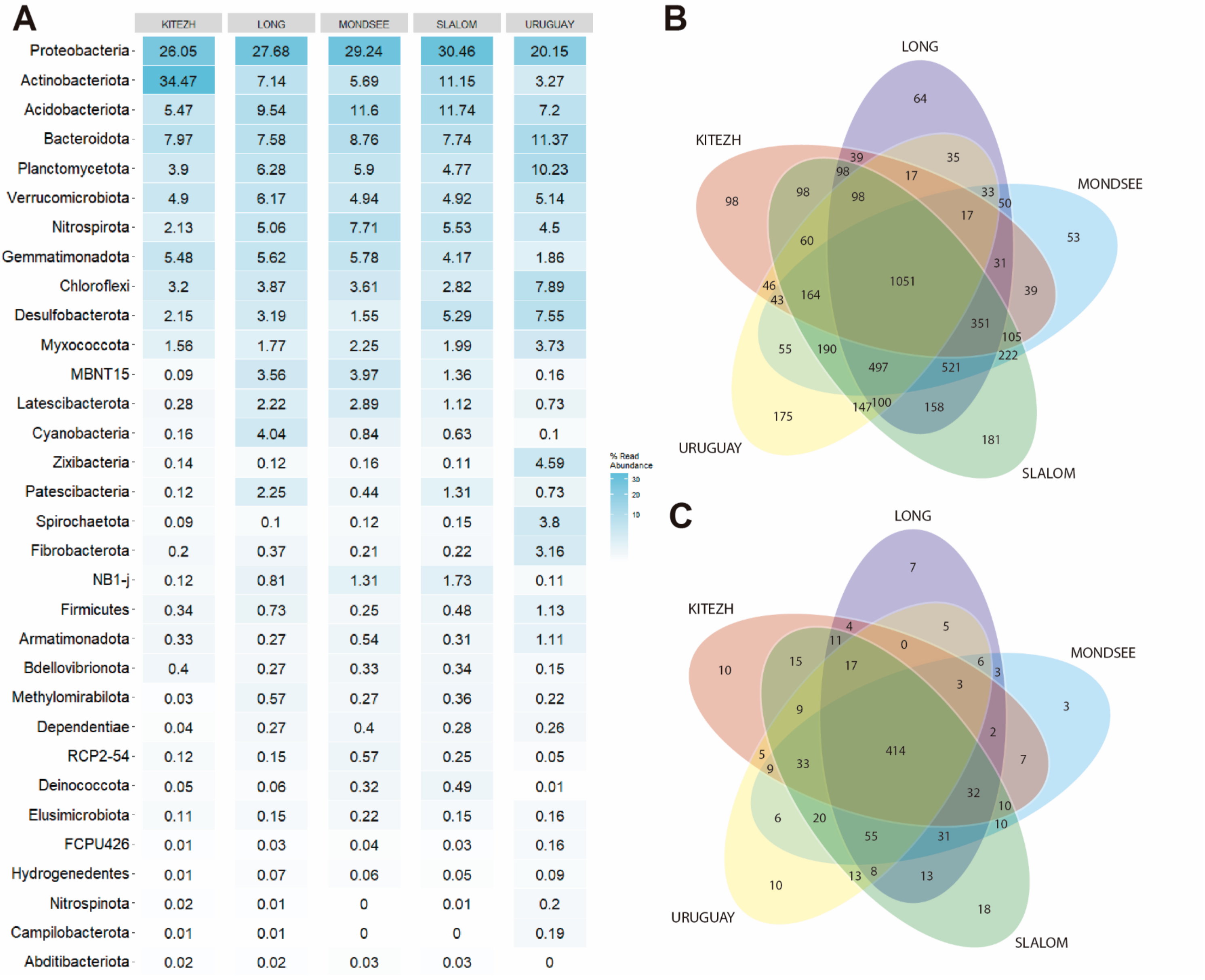

Supplementary Figure S3. Dendrogram obtained by clustering analysis with UPGMA algorithm, using Jaccard index for similarity measures (height values in the scale). Diversity at ASV level of total bacteria based on 16S rRNA gene analysis **(A)** and methane oxidizing bacteria based on *pmoA* gene analysis **(B)**.

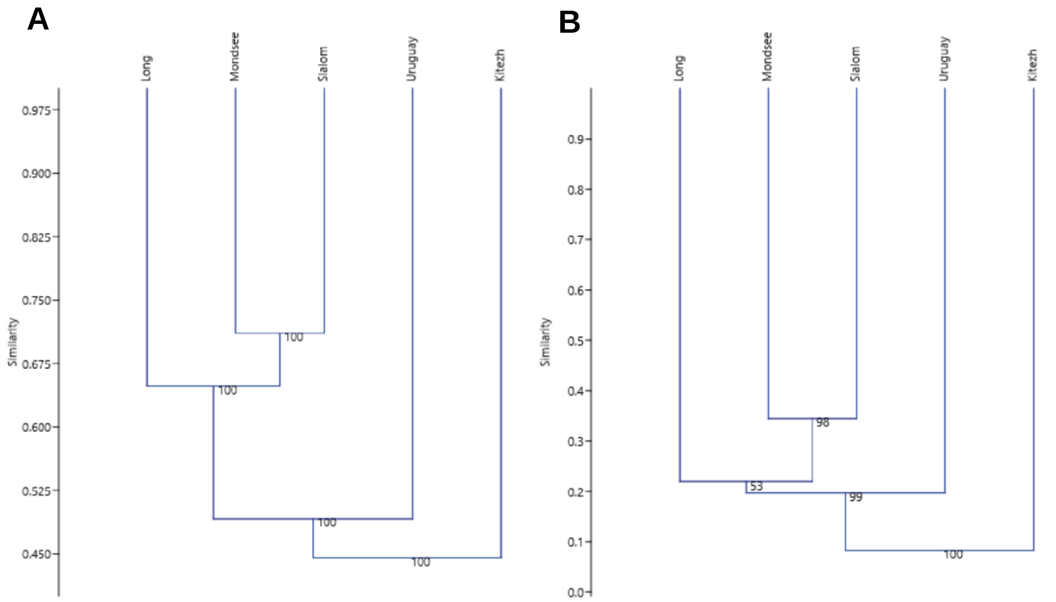

Supplementary Figure S4. Maximum likelihood tree of partial bacterial *pmoA* gene sequences and representative species of currently know methane oxidizing bacteria of *Gammaproteobacteria* class **(A)**, *Alphaproteobacteria* class **(B)** and *Verrucomicrobia* phylum **(C)**. Jukes Cantor model was used to calculate the evolutionary distance matrices. Bootstrap values (above 50%) based on 100 re-samplings are listed at the nodes. The scale bar represent 0.5 **(A** and **B)** and 0.2 substitutions per nucleotide position **(C)**

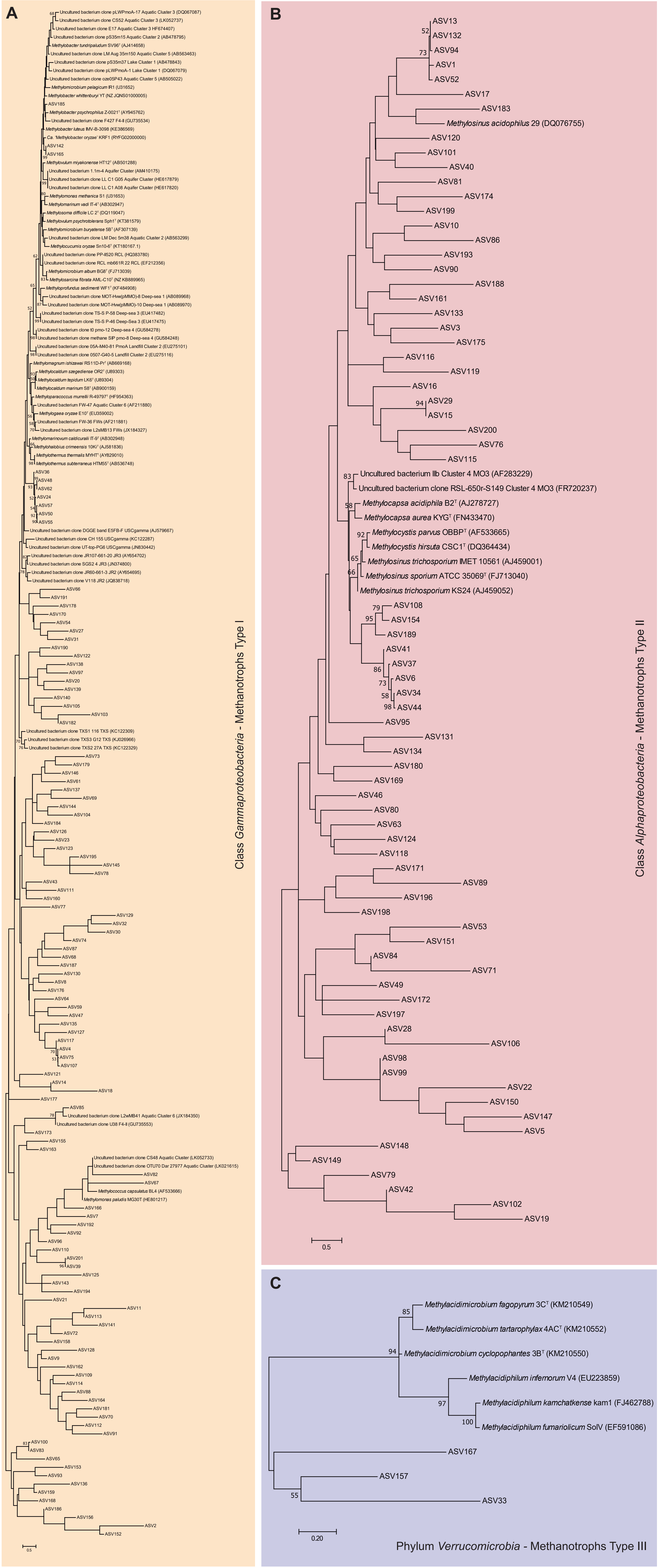

Supplement: Supplementary file 1 [file Data_Sheet_1.PDF]
